# Neurocognitive monitoring in patients undergoing opioid pain medication after spinal surgery: a feasibility study of a new monitoring method

**DOI:** 10.1007/s00701-023-05486-w

**Published:** 2023-01-10

**Authors:** Vicki Marie Butenschoen, Ferdinand Wriedt, Bernhard Meyer, Sandro M. Krieg

**Affiliations:** Department of Neurosurgery, Technical University Munich, School of Medicine, Klinikum Rechts Der Isar, Ismaningerstr. 22, 81675 Munich, Germany

**Keywords:** Cognitive impairment, Spine surgery, Digital health care

## Abstract

**Purpose:**

Patients undergoing spinal surgery require postoperative pain management to alleviate wound pain. Pain medication includes WHO grade 1 analgesic as well as potent opioids, potentially leading to cognitive decline. Up until now, the cognitive impairment is only poorly studied and difficult to monitor. We hereby investigate the feasibility of a digital monitoring method for neurocognitive function under opioid medication after spinal instrumentation.

**Methods:**

Prospective monocenter feasibility study enrolling patients before undergoing spinal surgery. We performed cognitive testing using a tablet-based application before (baseline), as well as on day 2 after surgery (intravenous opioids), before discharge (oral opioids), and at follow-up. We recorded the exact pain medication and its other side effects. Potential risk factors for the postoperative decline in cognition included age, high-dose opioid application, and length of surgery.

**Results:**

We included 20 patients in our study. The baseline assessment revealed no cognitive impairment before surgery. All patients underwent dorsal instrumentation for degenerative (60%), osteoporotic fracture (15%), or spinal tumor (25%) indications. Cognitive testing after surgery showed a significant decline under intravenous opioid therapy including short time and delayed verbal recall (*p* < 0.001) as well as arithmetic fluency. Cognitive performance significantly improved with partial recovery until follow-up and opioid discontinuation.

**Conclusion:**

Cognition testing and monitoring of neurocognitive decline under high-dose opioid medication were feasible using the digital tablet-based application. The cognition app helps to identify difficulties in cognitive function as a side effect of overdosage in opioid medication, and care givers should evaluate the risk of non-comprehension and impaired informed consent appropriately.

## Introduction

Patients undergoing spinal surgery require postoperative pain medication, and its intensity, dosage, and duration may depend on the extent of surgery [12] and individual factors [1, 3, 13]. Postoperative pain management includes non-steroidal anti-inflammatory drugs (NSAIDs), central analgesics (metamizole), and opioids ranging from a low (tilidine, tramadol) to a high analgetic potency (piritramide, hydromorphone, fentanyl) [18]. Scientific literature well describes the opioid side effects which include nausea, dizziness [9], respiratory depression [2], withdrawal symptoms and addiction [16], and cognitive decline [7, 8, 14, 15] up to sedation.

Considering cognitive testing presents a demanding assessment, data on postoperative decrease of cognition due to opioid administration in the postoperative phase in spine patients is sparse, and standardized testing without rater bias is lacking. Therefore, we aimed to assess postoperative cognitive decline due to postoperative pain management in an inpatient setting, comparing cognitive performance with baseline and follow-up examination.

## Methods

### Study design

We performed a single-center prospective cohort study at the Department of Neurosurgery at a tertiary care hospital in Munich, Germany. We asked all patients for their permission to participate prior to their surgical treatment and they signed an informed consent form before the data assessment. Ethical approval was obtained prior to patient enrolment. After enrolment, patients underwent cognitive testing using a tablet-based cognition app previously established in our department in glioma patients [4].

Inclusion criteria were over 18 years of age, planned spinal surgery for degenerative or trauma indication with planned hospital stay, and ability to agree to participate in our study and follow-up examinations. We excluded patients suffering from depression, dementia, or Parkinson’s disease, presuming their medication intake would restrain cognitive assessment.

Medication history and visual analog scale (VAS) scores were assessed before surgery (t0), after surgery (t1, piritramide intravenous (iV) ranging between 30, and 80 mg/24 h), t2 (oral opioids, including tramadol, or oxycodone) and t3 at follow-up examinations. Dose equivalents were assessed and expressed in oral morphine or iV morphine dose values per 24 h, depending on medication administration.

### Cognition app

**Fig. 1:**
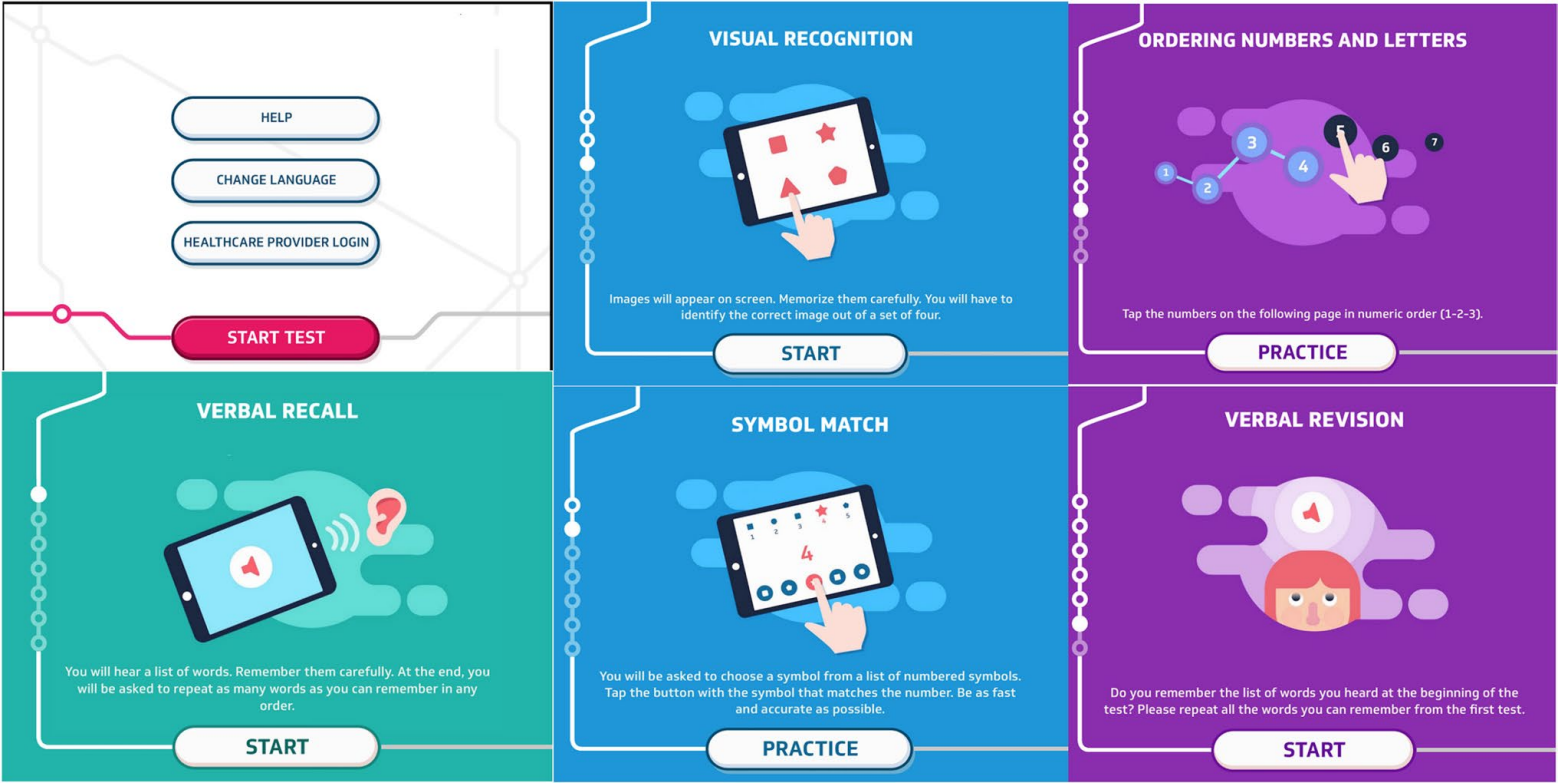
Illustrations of different cognitive tests in which the digitized app function is used, with instructions

We used a previously validated tablet-based mobile app to assess cognitive function in all patients [4]. The mobile cognition app comprises tests on memory, vocabulary, and logic; provides automated scoring and standardized instructions; and has been recently presented in our previous work [4] (Fig. 1). We performed cognitive assessment at four distinct appointments: baseline assessment before surgery (t0), cognitive testing on day 2 after surgery with intravenous opioid analgesics (t1), before discharge with oral opioid treatment (t2), and at the follow-up appointment (t3).

### Statistical analysis

We performed statistical analyses were performed using the SPSS software version 28.0.0.0 (IBM Corp.). We compared categorical data by applying the chi-square or Fisher’s exact test. The mean values were compared using the independent-samples *t*-test; the mean values of the test results were compared by applying the paired *t*-test. All tests were performed 2-sided, and a *p*-value less than 0.05 was considered significant.

### Ethical considerations

This study is in accordance with ethical standards outlined in the Declaration of Helsinki, and we obtained approval from our local ethics committee prior to subject recruitment (registration number 365/20 S-SR). All patients gave written informed consent prior to the cognitive testing.

## Results

### Patient population

In total, we prospectively enrolled 20 patients before surgery, with a median age of 67 years (range 36–84 years) between July 2021 and March 2022. Male and female patients were equally distributed (50%, *n* = 10 each).

All patients underwent a dorsal spinal instrumentation. Indications included degenerative spinal canal stenosis with previous decompression or spondylolisthesis (60%, *n* = 12), osteoporotic fractures with beginning deformity (15%, *n* = 3), and spinal instrumentation with carbon instrumentation for metastatic spinal disease (25%, *n* = 5).

Overall, three patients reported on a preoperative oral opioid intake with tramadol (one patient, 50 mg in the morning and evening), tilidine (one patient, 100 mg in the morning and evening), or Oxycodone/Naloxone (one patient, 10/5 mg retard, morning, and evening dose). Of the patients, 85% were not on any opioids before surgery. Table 1 shows patient demographics.

**Table 1:** Patient demographics including age, gender, length of surgery, and follow-up appointment (t3), indications for spinal instrumentation, as well as mean visual analog scale (VAS) score for pre- and postoperative pain

Age (years)	Median	67
	Minimum	36
	Maximum	84
Length of surgery	Mean	154
	Minimum	29
	Maximum	234
Gender	Female	50%
	Male	50%
Follow-up (days)	Median	72.5
	Minimum	30
	Maximum	279
Indication for surgery	Degenerative	60%
	Osteoporotic fracture	15%
	Metastases	25%
Pain (VAS-score)		
Before surgery	Mean	5.5
Directly after surgery	Mean	4.1
At discharge	Mean	2.7
At follow-up	Mean	0.5

### Cognitive assessment

**Fig. 2:**
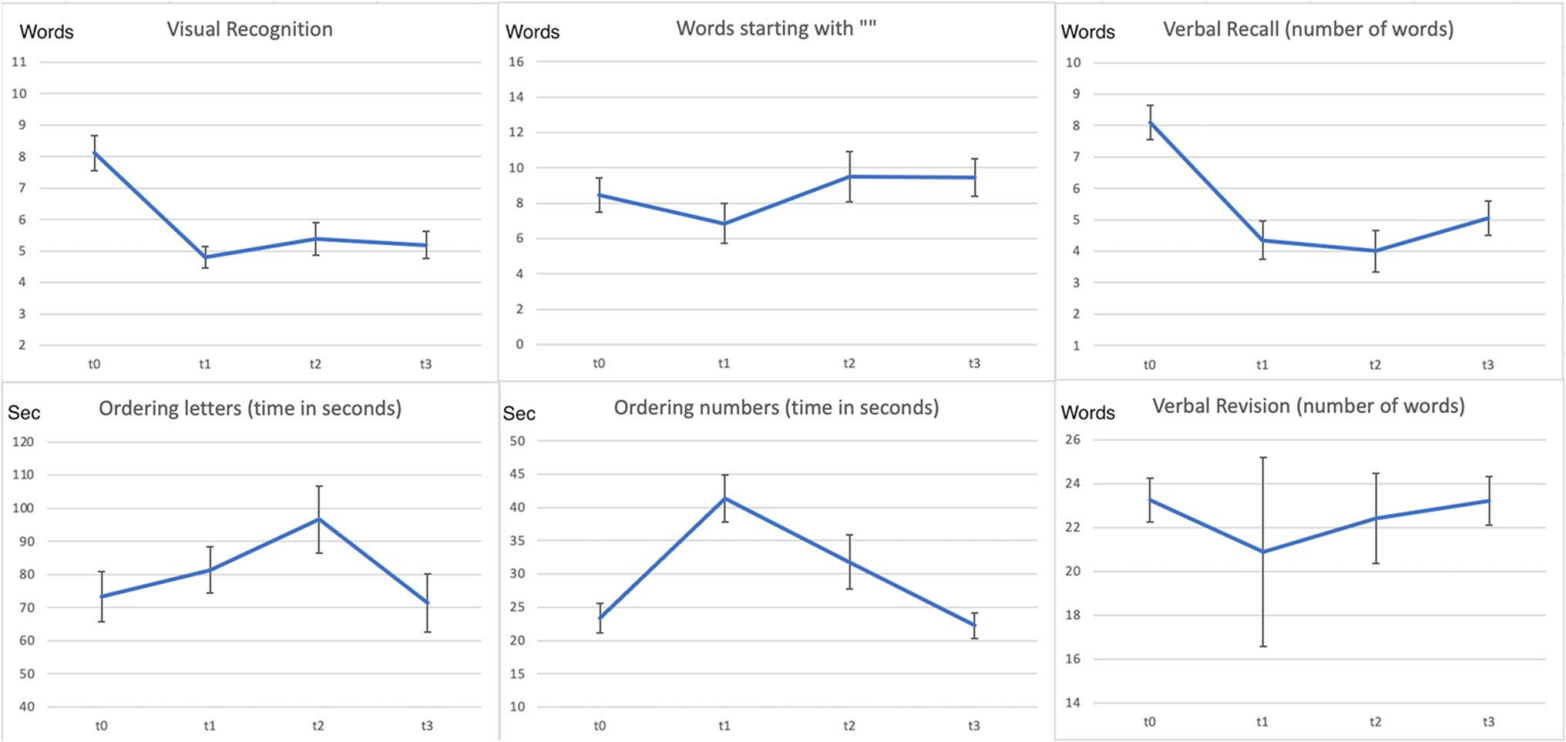
Test results (*y*-axis) at different timing (t0, t1, t2, and t3) showing cognitive decline under intravenous opioid medication and stepwise recovery until follow-up in most testing modalities. The plotted vertical error bars indicate the standard error of the mean

Complete preoperative, postoperative, and follow-up data assessment was available in all patients. We performed baseline cognitive testing at t0 with a median of 2 d (range 1 to 13 d) prior to surgical treatment. Median values and ranges for word memory, short and delayed recall, arithmetic fluency, and test durations are described in Table 2 and Fig. 2.

**Table 2:** Median (number of words for visual recognition, verbal recall, words that start with “” and ordering symbols) and mean (time) values for cognition tests performed at T0 (baseline before surgery), T1 (under iV medication), T2 (oral medication), and at follow-up examination (T3)

Median and mean values (SD)	Visual recognition	Verbal recall	Words that start with ""	Time ordering letters (s)	Time ordering numbers (s)	Ordering symbols	Time ordering symbols (s)	Verbal revision
T0 (baseline)	8.3 (2.4)	8 (2.4)	9 (4.2)	73.3 (34.2)	23.3 (9.8)	22.5 (8.4)	4.5 (1.7)	24 (1)
T1 (iV)	4.3 (2)	4 (2.7)	6 (5)	81.3 (31.5)	41.3 (15.7)	22.5 (16.3)	4.2 (1.3)	22.5 (5.4)
T2 (oral)	5.8 (2.3)	3.5 (2.9)	9 (6.3)	96 (45.6)	31.8 (18.3)	20.5 (6.3)	4.2 (1.2)	23 (2.1)
T3 (Follow-up)	5.3 (1.9)	5 (2.4)	9 (4.7)	71.3 (39.6)	22.6 (8.5)	23.5 (6.8)	4 (1.2)	24 (1.1)

Two days after surgery (t1), the cognitive assessment revealed a significant decrease under the iV opioid administration, with a persistent impairment during the in-hospital stay under oral opioid treatment (Table 2, Fig. 2). Furthermore, we observed longer test durations and difficulties encountered in word memory testing. At a median follow-up of 10 weeks (72 days), cognitive testing showed a recovery with patients reaching the preoperative baseline results in most categories. At the follow-up examination, three patients continued a reduced opioid medication (15%).

Not all cognitive performances showed the same differences with opioid administration. The most sensitive differences were tested for word memory (remembering words *p* < 0.001, repeating words *p* < 0.001). The verbal revision had a clear trend toward lower performance scores but failed to reach statistical significance due to only small differences (*p* = 0.055).

### Risk factors

With univariate analysis, age and male sex were not associated with postoperative cognitive deterioration regarding visual recognition and verbal revision (*p* = 0.792 and *p* = 0.454, respectively) but we identified a significant association between age and verbal recall (*p* = 0.025, *r* = 0.378). Interestingly, age affected overall test durations (duration of ordering letters, numbers, and symbols, *p* = 0.008, *r* = 0.681), but not the effects on cognition after opioid medication (*p* = 0.171).

In multivariate analysis, age remained a significant contributing factor for the absolute postoperative test results in the category verbal recall (*p* = 0.031 for postoperative and *p* = 0.023 before discharge) when controlling for sex and indications for surgery. We did not identify any factors contributing to the postoperative deterioration (relative decline).

When analyzing the actual cognitive decline, the intravenous dosage of piritramide administration did not significantly affect the test results. The preoperative intake of opioids did not influence the presence or the quantification of the cognitive decline under IV medication (visual recognition *p* = 0.935 and recall *p* = 0.681). Patients suffering from osteoporotic fractures had a stronger decline compared to patients suffering from degenerative or metastatic processes, but the association failed to reach statistical significance.

The length of surgery did not influence the quantified cognitive decline after surgery.

## Discussion

In our patient cohort, cognitive assessment of patients undergoing spinal instrumentation was feasible. We found a significant reduction of cognitive abilities under intravenous opioid administration with a recovery at follow-up appointments.

Our study has several limitations. A first and important limitation to mention is the non-assessable role of anesthesia during the operative procedure. Postoperative neurocognitive disorders after general anesthesia and surgery have been described [5, 17], and its share in the overall decline after surgery is unclear. Considering all patients underwent opioid treatment in our department, we cannot control for one or the other factors such as pain, fear, or response to stress, and we consider cognitive deterioration a multifactorial problem after surgery. Another possibly confounding factor was pain itself. Pain may lead to cognitive decline and reduced attentional functioning in patients [10] and it makes the differentiation between pain-related and treatment-related cognitive impairment very difficult. Despite these limitations, the overall cognitive assessment was feasible in all patients.

Another limitation is the potential learning effect as a result of repeated tests which may bias the results at discharge and follow-up analysis. Yet, we saw a decline after surgery, and follow-up testing was performed after a median of 10 weeks.

With 20 patients included in our study, the sample size is rather small; yet it is a pilot study. We started our prospective assessment as a feasibility study. Further prospective studies including the digital cognitive testing may allow us more analyses of risk factors and influencing factors. Considering the digital cognitive testing is completely rater-independent and standardized, multicenter studies are possible and planned for further investigations.

Data privacy remains a critical aspect in digital health assessment. Protecting sensible data, such as digital results, is crucial, and treating physician must secure and store postoperative cognitive impairment information. If stored otherwise, patients must be informed, and they must provide their written consent before the assessment begins.

Not all cognitive abilities showed the same tendency of decline under opioid treatment. We found a strong association between opioid dosage and memory testing (verbal recall and visual recognition) as well as vocabulary (words starting with “E”). In contrast, arithmetic fluency remained stable during the time of testing with only a minimum non-significant decline. Furthermore, test durations of arithmetic fluency showed a tendency for slower cognitive processing. These results may implicate a disturbed ability to recall, and process presented complications and risks of further operative treatments under intravenous opioid administration and patients should be informed more individually and patiently.

The potential of digital cognitive function assessment in patients under pain medication is certainly not limited to neurosurgical patients undergoing spinal instrumentations. Cognitive side effects affect chronic pain patients undergoing long-term opioid analgesics [14, 15] and may offer a reliable tool to estimate and prevent long-term opioid overdosing in the outpatient setting without requiring more health care resources. In times of healthcare digitalization, a digital assessment tool allows improved access to follow-up monitoring and rater-independent patient monitoring [11]. Test results may present a visualization of burdening side effects on a large time scale, and they be visible to the caregiver as well as the caretaker. Furthermore, outpatient monitoring presents an important component of enhanced recovery after surgery protocols and may facilitate shorter hospital stays including follow-up pain management at home [6].

## Conclusion

Overall, cognition testing and monitoring of cognitive decline under high-dose opioid medication were feasible using the digital application. Therefore, the tablet-based approach helps to identify difficulties in cognitive function as a side effect of overdosage in opioid medication, and care givers should evaluate the risk of non-comprehension after a surgical treatment appropriately.

## Data Availability

The datasets used and/or analyzed during the current study are available from the corresponding author on reasonable request.
